# Rietveld refinement of the langbeinite-type phosphate K_2_Ni_0.5_Hf_1.5_(PO_4_)_3_

**DOI:** 10.1107/S2056989020012062

**Published:** 2020-09-11

**Authors:** Liang Zhou, Denys S. Butenko, Ivan V. Ogorodnyk, Nickolai I. Klyui, Igor V. Zatovsky

**Affiliations:** a College of Physics, Jilin University 2699 Qianjin St., 130012 Changchun, People’s Republic of China; b ShimUkraine LLC, 18, Chigorina Str., office 429, 01042 Kyiv, Ukraine; c V. Lashkaryov Institute of Semiconductor Physics, NAS of Ukraine, 41 Pr. Nauki, 03028 Kyiv, Ukraine

**Keywords:** powder diffraction, langbeinite structure type, multimetal phosphate, crystal structure

## Abstract

Polycrystalline potassium nickel(II) hafnium(IV) tris­(orthophosphate), a langbeinite-type phosphate, was synthesized by a solid-state method. The three-dimensional framework of the title compound is built up from two types of [*M*O_6_] octa­hedra [the *M* sites are occupied by Hf:Ni in ratios of 0.754 (8):0.246 (8) and 0.746 (8):0.254 (8), respectively] and [PO_4_] tetra­hedra are connected *via* O vertices. The K^+^ cations are located in two positions within large cavities of the framework, having coordination numbers of 9 and 12. The Hf, Ni and K sites lie on threefold rotation axes, while the P and O atoms are situated in general positions.

## Abstract 2

Cubic K_2_Ni_0.5_Hf_1.5_(PO_4_)_3_ crystallizes in the langbeinite structure type. The principal building units are two independent [(Ni,Hf)O_6_] octa­hedra, [PO_4_] tetra­hedra and [KO_9_] and [KO_12_] polyhedra.

## Chemical context   

Langbeinite-related complex oxides have a variety of inter­esting properties, for example, ferroelectricity or ferroelasticity (Norberg, 2002 ▸). In particular, complex phosphates of this type have attracted attention for their high thermal and chemical stability, and many different combinations for structural substitutions are possible (Wulff *et al.*, 1992 ▸; Slobodyanik *et al.*, 2012 ▸). These characteristics made it possible to propose the family of langbeinite-type phosphates as successful hosts for the immobilization of radioactive waste (Orlova *et al.*, 2011 ▸). Moreover, in the last decade rare-earth (RE)-containing langbeinite-type phosphates have been studied intensively owing to their outstanding luminescent properties and applications in LEDs (Liang & Wang, 2011 ▸; Liu *et al.*, 2016 ▸; Sadhasivam *et al.*, 2017 ▸; Terebilenko *et al.*, 2020 ▸). Accordingly, further studies of iso- and heterovalent substitution within the cationic sites of the langbeinite structure are important. Structural data for langbeinite-type Hf-containing phosphates are scarce and include only K_1.93_Mn_0.53_Hf_1.47_(PO_4_)_3_ (Ogorodnyk *et al.*, 2007*a* ▸) and K_2_YHf(PO_4_)_3_ (Ogorodnyk *et al.*, 2009 ▸).

In this report, we describe the powder X-ray refinement using the Rietveld method for the multimetal phosphate K_2_Ni_0.5_Hf_1.5_(PO_4_)_3_ (**I**), structurally isotypic with the mineral langbeinite, K_2_Mg_2_(SO_4_)_3_ (Zemann & Zemann, 1957 ▸).

## Structural commentary   

**Figure 1:**
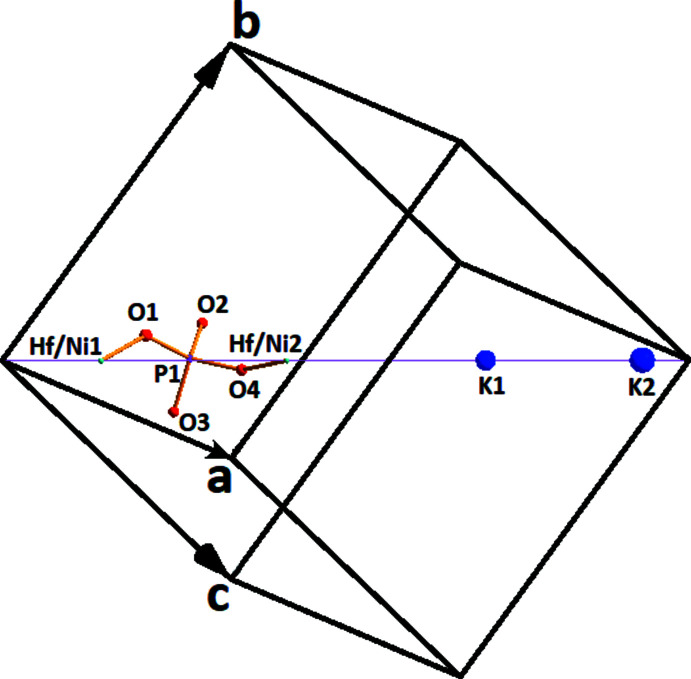
A view of the asymmetric unit of K_2_Ni_0.5_Hf_1.5_(PO_4_)_3_, with displacement spheres drawn at the 50% probability level.

As shown in Fig. 1 ▸, in the structure of (**I**) the K, Ni and Hf sites are localized on threefold rotation axes (Wyckoff position 4 *a*), while the P and all O atoms occupy general sites (12 *b*). Two metallic sites (Hf,Ni)1 and (Hf,Ni)2 show mixed occupancy with a Hf:Ni ratio of about 0.75:0.25 (nickel proportion 0.246 (8) for the *M*1 site and 0.254 (8) for the *M*2 site). A similar *M* ^II^:*M* ^IV^ ratio was also observed for isostructural phosphates of general composition *M* ^I^ *M* ^II^_0.5_ *M* ^IV^_1.5_(PO_4_)_3_, *viz*. K_2_Ni_0.5_Ti_1.5_(PO_4_)_3_ (Ogorodnyk *et al.*, 2007*b* ▸), Rb_2_Ni_0.5_Ti_1.5_(PO_4_)_3_ (Strutynska *et al.*, 2015 ▸), K_2_Co_0.5_Ti_1.5_(PO_4_)_3_ and K_2_Mn_0.5_Ti_1.5_(PO_4_)_3_ (Ogorodnyk *et al.*, 2006 ▸), K_2_Ni_0.5_Zr_1.5_(PO_4_)_3_ (Zatovsky, 2014 ▸), K_1.96_Mn_0.57_Zr_1.43_(PO_4_)_3_ and K_1.93_Mn_0.53_Hf_1.47_(PO_4_)_3_ (Ogorodnyk *et al.*, 2007*a* ▸).

**Figure 2:**
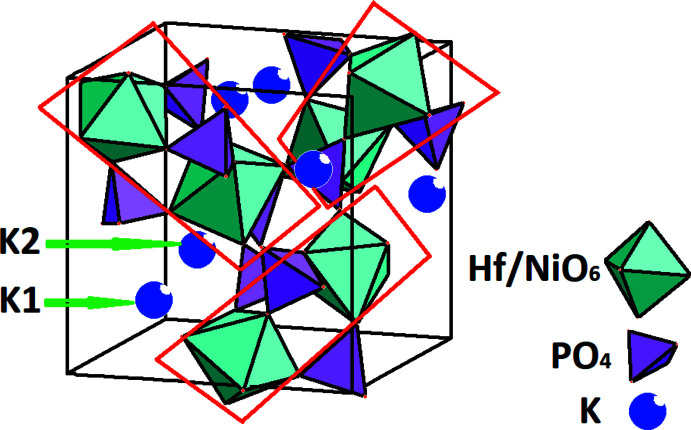
[*M*_2_P_3_O_18_] building unit (highlighted in red frames) for (**I**). K^+^ cations are shown as blue spheres of arbitrary radius.

**Figure 3:**
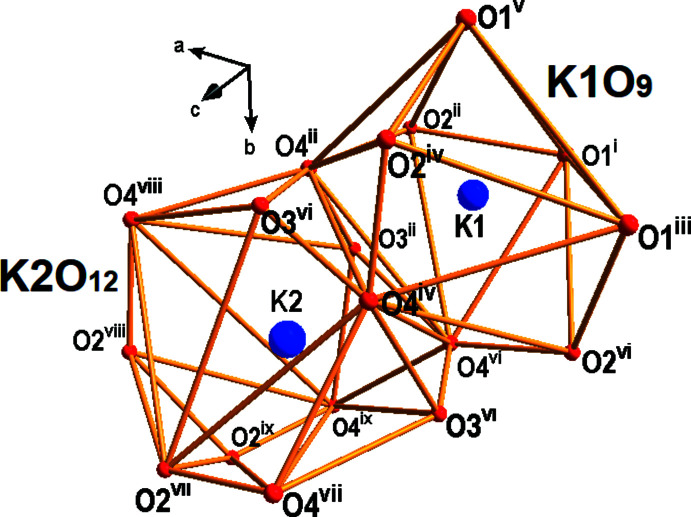
Coordination polyhedra [K1O_9_] and [K2O_12_] for (**I**). Displacement spheres are drawn at the 50% probability level. [Symmetry codes: (i) −*x* + 1, *y* + 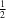, −*z* + 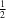; (ii) −*x* + 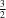, −*y* + 1, *z* + 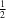; (iii) −*z* + 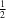, −*x* + 1, *y* + 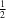; (iv) −*y* + 1, *z* + 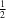, −*x* + 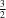; (v) *y* + 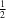, −*z* + 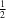, −*x* + 1; (vi) *z* + 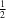, −*x* + 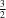, −*y* + 1; (vii) −*z* + 1, *x* + 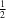, −*y* + 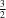; (viii) −*y* + 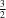, −*z* + 1, *x* + 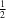; (ix) *x* + 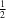, −*y* + 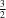, −*z* + 1].

The (Hf,Ni)—O distances in (**I**) are 1.989 (15) and 2.121 (14) Å for the [(Hf,Ni)1O_6_] octa­hedron, and 2.131 (17) and 2.172 (16) Å for the [(Hf,Ni)2O_6_] octa­hedron. The two independent [(Hf,Ni)O_6_] octa­hedra are linked by three [PO_4_] tetra­hedra to form an [*M*_2_P_3_O_18_] building unit (Fig. 2 ▸). These building units are arranged along three directions (threefold rotation axes) and linked together *via* oxygen vertices, forming a three-dimensional framework structure. Pairs of K^+^ cations (two independent sites) are localized in large cavities of the resulting framework. The potassium cations are found in 9- and 12-coordination by O atoms with K—O distances ranging from 2.854 (17) Å to 3.372 (18) Å (Table 1 ▸, Fig. 3 ▸), leading to distorted polyhedra. The [PO_4_] tetra­hedron shows considerable distortion (Table 1 ▸).

For (**I**), the calculation of BVS (bond-valence sums) was performed using the parameters for Hf from Brese & O’Keeffe (1991 ▸), for Ni from Brown (private communication, 2001 ▸) and for K, P from Brown & Altermatt (1985 ▸). The corresponding occupation of the *M* sites by Hf and Ni atoms was taken into account. The sum of BVS of the cations is +23.67 valence units (v.u.), which is close to the −24 v.u. required for the O atoms.

## Synthesis and crystallization   

**Figure 4:**
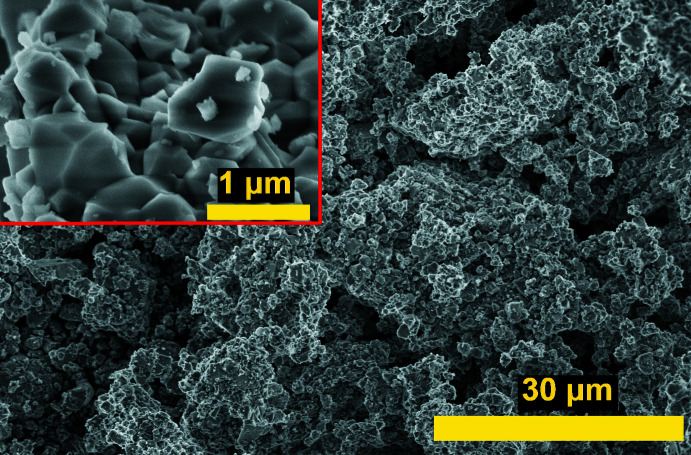
SEM image for (**I**) (Insert: image at higher magnification).

Compound (**I**) was synthesized using a solid-state reaction method. A well-ground starting mixture of 3.157 g HfO_2_, 0.374 g NiO, 2.361 g KPO_3_ and 1.150 g NH_4_H_2_PO_4_ (molar ratio K:Ni:Hf:P = 4:1:3:6) was transferred to a ceramic crucible and pre-heated at 553 K for 2 h. The powder was re-ground, heated at 823 K for 3 h and then milled for 0.5 h in an agate mortar. The resulting fine powder was pressed into a pill and finally calcined at 1273 K for 100 h. The sample was ground before performing powder XRD data collection. Scanning electron microscopy (SEM, Magellan 400, recorded at 10 kV) showed that the obtained sample is an aggregate of small crystallites with a size less than 1 µm (Fig. 4 ▸).

## Refinement   

**Figure 5:**
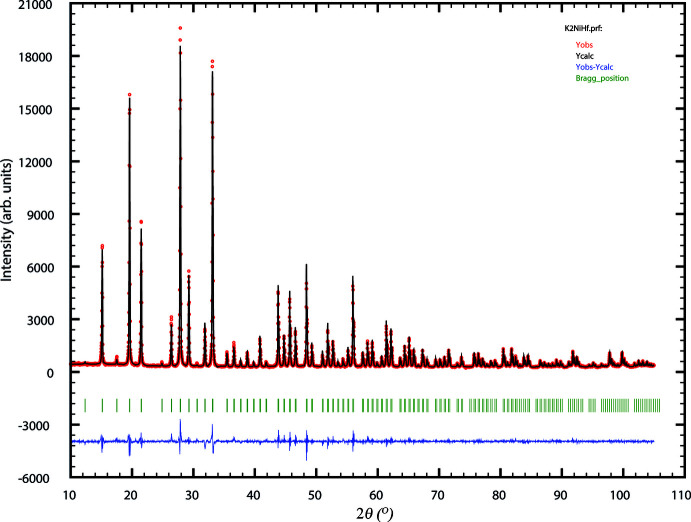
Rietveld refinement of K_2_Ni_0.5_Hf_1.5_(PO_4_)_3_. Experimental (dots), calculated (red curve) and difference (blue curve) data for 2*θ* range 10–108°.

The experimental, calculated and difference pattern are shown in Fig. 5 ▸. Crystal data, data collection and structure refinement details are summarized in Table 2 ▸. Structure refinement was performed using K_2_YHf(PO_4_)_3_ (Ogorodnyk *et al.*, 2009 ▸) as a starting model. A modified pseudo-Voigt function (Thompson *et al.*, 1987 ▸) was used for the profile refinement. The similar shape of the transition-metal octa­hedra indicated that both *M* positions are occupied by Ni and Hf simultaneously. For the refinement of their occupancies their coordinates and *U*_iso_ values were constrained together, and the sum of occupancies constrained to unity for both sites.

## Supplementary Material

Crystal structure: contains datablock(s) global, I. DOI: 10.1107/S2056989020012062/wm5581sup1.cif

Rietveld powder data: contains datablock(s) I. DOI: 10.1107/S2056989020012062/wm5581Isup2.rtv

CCDC reference: 2026681

Additional supporting information:  crystallographic information; 3D view; checkCIF report

## Figures and Tables

**Table 1:** Selected geometric parameters (Å, °)

K1—O1^i^	2.854 (17)	K2—O4^iii^	3.372 (18)
K1—O4^ii^	3.082 (17)	P1—O1	1.503 (15)
K1—O2^ii^	3.103 (15)	P1—O2	1.533 (17)
K2—O3^ii^	2.944 (16)	P1—O3	1.48 (2)
K2—O2^iii^	2.987 (18)	P1—O4	1.506 (18)
K2—O4^ii^	3.041 (18)		
			
O1—P1—O2	110.2 (10)	O2—P1—O3	112.6 (10)
O1—P1—O3	107.4 (10)	O2—P1—O4	106.0 (10)
O1—P1—O4	120.1 (10)	O3—P1—O4	100.3 (11)

Symmetry codes: (i) 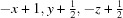; (ii) 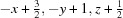; (iii) 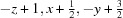.

**Table 2:** Experimental details

Crystal data	
Chemical formula	K_2_Ni_0.5_Hf_1.5_(PO_4_)_3_
*M*_r_	660.19
Crystal system, space group	Cubic, *P*2_1_3
Temperature (K)	293
*a* (Å)	10.12201 (5)
*V* (Å^3^)	1037.05 (1)
*Z*	4
Radiation type	Cu *K*α_1_, λ = 1.540598 Å
Specimen shape, size (mm)	Flat sheet, 15 × 15

Data collection	
Diffractometer	Haoyuan Instrument Co. Ltd DX-2700B
Specimen mounting	Glass container
Data collection mode	Reflection
Scan method	Step
2θ values (°)	2θ_min_ = 10.008 2θ_max_ = 105.008 2θ_step_ = 0.020

Refinement	
*R* factors and goodness of fit	*R*_p_ = 6.111, *R*_wp_ = 7.831, *R*_exp_ = 4.020, *R*_Bragg_ = 4.709, *R*(*F*) = 3.21, χ^2^ = 4.410
No. of parameters	107
No. of restraints	3

Computer programs: data-collection and reduction software supplied by instrument manufacturer (http://www.haoyuanyiqi.com/en/xsxysy/s_23_30.html), *FULLPROF* (Rodriguez-Carvajal, 2020 ▸), *DIAMOND* (Brandenburg, 2006 ▸), *PLATON* (Spek, 2020 ▸), *WinGX* (Farrugia, 2012 ▸) and *enCIFer* (Allen *et al.*, 2004 ▸).

## supplementary crystallographic information

### Crystal data

**Table:**

K_2_Ni_0.5_Hf_1.5_(PO_4_)_3_	*D*_x_ = 4.228 Mg m^−^^3^
*M**_r_* = 660.19	Cu *K*α radiation, λ = 1.540598 Å
Cubic, *P*2_1_3	*T* = 293 K
Hall symbol: P 2ac 2ab 3	Particle morphology: tetrahedra
*a* = 10.12201 (5) Å	yellow
*V* = 1037.05 (1) Å^3^	flat_sheet, 15 × 15 mm
*Z* = 4	Specimen preparation: Prepared at 293 K and 101.3 kPa

### Data collection

**Table:**

Haoyuan Instrument Co. Ltd DX-2700B diffractometer	Data collection mode: reflection
Radiation source: X-ray tube, X-ray	Scan method: step
Graphite monochromator	2θ_min_ = 10.008°, 2θ_max_ = 105.008°, 2θ_step_ = 0.020°
Specimen mounting: glass container	

### Refinement

**Table:**

*R*_p_ = 6.111	107 parameters
*R*_wp_ = 7.831	3 restraints
*R*_exp_ = 4.020	3 constraints
*R*_Bragg_ = 4.709	Standard least squares refinement
*R*(*F*) = 3.21	(Δ/σ)_max_ = 0.001
4751 data points	Background function: Linear Interpolation between a set background points with refinable heights
Profile function: Thompson-Cox-Hastings pseudo-Voigt * Axial divergence asymmetry	Preferred orientation correction: Modified March's Function

### Fractional atomic coordinates and isotropic or equivalent isotropic displacement parameters (Å^2^)

**Table:**

	*x*	*y*	*z*	*U*_iso_*/*U*_eq_	Occ. (<1)
K1	0.7042 (5)	0.7042 (5)	0.7042 (5)	0.028 (4)*	
K2	0.9319 (8)	0.9319 (8)	0.9319 (8)	0.044 (4)*	
Ni1	0.14423 (16)	0.14423 (16)	0.14423 (16)	0.0022 (12)*	0.246 (8)
Ni2	0.4147 (2)	0.4147 (2)	0.4147 (2)	0.0019 (12)*	0.254 (8)
Hf1	0.14423 (16)	0.14423 (16)	0.14423 (16)	0.0022 (12)*	0.754 (8)
Hf2	0.4147 (2)	0.4147 (2)	0.4147 (2)	0.0019 (12)*	0.746 (8)
P1	0.4624 (6)	0.2349 (10)	0.1229 (9)	0.004 (2)*	
O1	0.3218 (13)	0.2314 (17)	0.0752 (16)	0.011 (6)*	
O2	0.5508 (14)	0.3023 (16)	0.0201 (15)	0.008 (4)*	
O3	0.5028 (13)	0.0973 (17)	0.1500 (18)	0.008 (6)*	
O4	0.4953 (16)	0.2985 (17)	0.2533 (14)	0.009 (6)*	

### Atomic displacement parameters (Å^2^)

**Table:**

	*U*^11^	*U*^22^	*U*^33^	*U*^12^	*U*^13^	*U*^23^
?	?	?	?	?	?	?

### Geometric parameters (Å, º)

**Table:**

K1—O1^i^	2.854 (17)	Hf1—O1	2.121 (14)
K1—O4^ii^	3.082 (17)	Hf1—O2^x^	1.989 (15)
K1—O2^ii^	3.103 (15)	Hf1—O1^xi^	2.121 (14)
K1—O1^iii^	2.854 (17)	Hf1—O2^xii^	1.989 (15)
K1—O4^iv^	3.082 (17)	Hf2—O4	2.172 (16)
K1—O2^iv^	3.103 (15)	Hf2—O3^i^	2.131 (17)
K1—O1^v^	2.854 (17)	Hf2—O4^xi^	2.172 (16)
K1—O4^vi^	3.082 (17)	Hf2—O3^iii^	2.131 (17)
K1—O2^vi^	3.103 (15)	Ni1—O1	2.121 (14)
K2—O3^ii^	2.944 (16)	Ni1—O2^x^	1.989 (15)
K2—O2^vii^	2.987 (18)	Ni1—O1^xi^	2.121 (14)
K2—O4^ii^	3.041 (18)	Ni1—O2^xii^	1.989 (15)
K2—O4^vii^	3.372 (18)	Ni2—O4	2.172 (16)
K2—O3^iv^	2.944 (16)	Ni2—O3^i^	2.131 (17)
K2—O2^viii^	2.987 (18)	Ni2—O4^xi^	2.172 (16)
K2—O4^iv^	3.041 (18)	Ni2—O3^iii^	2.131 (17)
K2—O4^viii^	3.372 (18)	P1—O1	1.503 (15)
K2—O3^vi^	2.944 (16)	P1—O2	1.533 (17)
K2—O2^ix^	2.987 (18)	P1—O3	1.48 (2)
K2—O4^vi^	3.041 (18)	P1—O4	1.506 (18)
K2—O4^ix^	3.372 (18)		
			
O1—Hf1—O2^x^	90.8 (6)	O1^xi^—Ni1—O2^x^	87.8 (6)
O1—Hf1—O1^xi^	93.7 (6)	O2^x^—Ni1—O2^xii^	87.7 (6)
O1—Hf1—O2^xii^	175.2 (6)	O1^xi^—Ni1—O2^xii^	90.8 (6)
O1^xi^—Hf1—O2^x^	87.8 (6)	O3^i^—Ni2—O4	95.2 (6)
O2^x^—Hf1—O2^xii^	87.7 (6)	O4—Ni2—O4^xi^	94.5 (6)
O1^xi^—Hf1—O2^xii^	90.8 (6)	O3^iii^—Ni2—O4	168.5 (6)
O3^i^—Hf2—O4	95.2 (6)	O3^i^—Ni2—O4^xi^	78.6 (6)
O4—Hf2—O4^xi^	94.5 (6)	O3^i^—Ni2—O3^iii^	92.7 (6)
O3^iii^—Hf2—O4	168.5 (6)	O3^iii^—Ni2—O4^xi^	95.2 (6)
O3^i^—Hf2—O4^xi^	78.6 (6)	O1—P1—O2	110.2 (10)
O3^i^—Hf2—O3^iii^	92.7 (6)	O1—P1—O3	107.4 (10)
O3^iii^—Hf2—O4^xi^	95.2 (6)	O1—P1—O4	120.1 (10)
O1—Ni1—O2^x^	90.8 (6)	O2—P1—O3	112.6 (10)
O1—Ni1—O1^xi^	93.7 (6)	O2—P1—O4	106.0 (10)
O1—Ni1—O2^xii^	175.2 (6)	O3—P1—O4	100.3 (11)

Symmetry codes: (i) −*x*+1, *y*+1/2, −*z*+1/2; (ii) −*x*+3/2, −*y*+1, *z*+1/2; (iii) −*z*+1/2, −*x*+1, *y*+1/2; (iv) −*y*+1, *z*+1/2, −*x*+3/2; (v) *y*+1/2, −*z*+1/2, −*x*+1; (vi) *z*+1/2, −*x*+3/2, −*y*+1; (vii) −*z*+1, *x*+1/2, −*y*+3/2; (viii) −*y*+3/2, −*z*+1, *x*+1/2; (ix) *x*+1/2, −*y*+3/2, −*z*+1; (x) *x*−1/2, −*y*+1/2, −*z*; (xi) *z*, *x*, *y*; (xii) −*z*, *x*−1/2, −*y*+1/2.

## References

[bb1] Allen, F. H., Johnson, O., Shields, G. P., Smith, B. R. & Towler, M. (2004). *J. Appl. Cryst.* **37**, 335–338.

[bb2] Brandenburg, K. (2006). *DIAMOND*. Crystal Impact GbR, Bonn, Germany.

[bb3] Brese, N. E. & O’Keeffe, M. (1991). *Acta Cryst.* B**47**, 192–197.

[bb4] Brown, I. D. (2001). Private communication.

[bb5] Brown, I. D. & Altermatt, D. (1985). *Acta Cryst.* B**41**, 244–247.

[bb6] Farrugia, L. J. (2012). *J. Appl. Cryst.* **45**, 849–854.

[bb7] Liang, W. & Wang, Y. (2011). *Mater. Chem. Phys.* **127**, 170–173.

[bb8] Liu, J., Duan, X., Zhang, Y., Li, Z., Yu, F. & Jiang, H. (2016). *J. Alloys Compd.* **660**, 356–360.

[bb9] Norberg, S. T. (2002). *Acta Cryst.* B**58**, 743–749. 10.1107/s0108768102013782 PMC2391006 12324686

[bb10] Ogorodnyk, I. V., Zatovsky, I. V., Baumer, V. N., Slobodyanik, N. S., Shishkin, O. V. & Vorona, I. P. (2007*a*). *J. Solid State Chem.* **180**, 2838–2844.

[bb11] Ogorodnyk, I. V., Zatovsky, I. V. & Slobodyanik, N. S. (2007*b*). *Russ. J. Inorg. Chem.* **52**, 121–125.

[bb12] Ogorodnyk, I. V., Zatovsky, I. V. & Slobodyanik, N. S. (2009). *Acta Cryst.* E**65**, i63–i64. 10.1107/S1600536809027573 PMC2977454 21583298

[bb13] Ogorodnyk, I. V., Zatovsky, I. V., Slobodyanik, N. S., Baumer, V. N. & Shishkin, O. V. (2006). *J. Solid State Chem.* **179**, 3461–3466.

[bb14] Orlova, A. I., Koryttseva, A. K. & Loginova, E. E. (2011). *Radiochemistry*, **53**, 51–62.

[bb15] Rodriguez-Carvajal, J. (2020). *FULLPROF*. Laboratoire Léon Brillouin (CEA–CNRS), France.

[bb16] Sadhasivam, S., Manivel, P., Jeganathan, K., Jayasankar, C. K. & Rajesh, N. P. (2017). *Mater. Lett.* **188**, 399–402.

[bb17] Slobodyanik, N. S., Terebilenko, K. V., Ogorodnyk, I. V., Zatovsky, I. V., Seredyuk, M., Baumer, V. N. & Gütlich, P. (2012). *Inorg. Chem.* **51**, 1380–1385. 10.1021/ic201575v 22260084

[bb18] Spek, A. L. (2020). *Acta Cryst.* E**76**, 1–11. 10.1107/S2056989019016244 PMC6944088 31921444

[bb19] Strutynska, N. Yu., Bondarenko, M. A., Ogorodnyk, I. V., Zatovsky, I. V., Slobodyanik, N. S., Baumer, V. N. & Puzan, A. N. (2015). *Cryst. Res. Technol.* **50**, 549–555.

[bb20] Terebilenko, K. V., Nedilko, S. G., Chornii, V. P., Prokopets, V. M., Slobodyanik, M. S. & Boyko, V. V. (2020). *RSC Adv.* **10**, 25763–25772. 10.1039/d0ra04975a PMC9055308 35518574

[bb21] Thompson, P., Cox, D. E. & Hastings, J. B. (1987). *J. Appl. Cryst.* **20**, 79–83.

[bb22] Wulff, H., Guth, U. & Loescher, B. (1992). *Powder Diffr.* **7**, 103–106.

[bb23] Zatovsky, I. V. (2014). *Acta Cryst.* E**70**, i41. 10.1107/S1600536814013658 PMC4120588 25161511

[bb24] Zemann, A. & Zemann, J. (1957). *Acta Cryst.* **10**, 409–413.

